# Serum branch-chained amino acids are increased in type 2 diabetes and associated with atherosclerotic cardiovascular disease

**DOI:** 10.1186/s12933-023-01958-6

**Published:** 2023-09-14

**Authors:** Juan Moreno-Vedia, Dídac Llop, Ricardo Rodríguez-Calvo, Núria Plana, Núria Amigó, Roser Rosales, Yaiza Esteban, Josefa Girona, Lluís Masana, Daiana Ibarretxe

**Affiliations:** 1 Vascular Medicine and Metabolism Unit, Research Unit on Lipids and Atherosclerosis, Sant Joan University Hospital, Universitat Rovira I Virgili, Institut Investigació Sanitaria Pere Virgili (IISPV), Reus, Spain; 2 Spanish Biomedical Research Centre in Diabetes and Associated Metabolic Disorders (CIBERDEM), Madrid, Spain; 3 Biosfer Teslab SL., Reus, Spain

## Abstract

**Background and aim:**

Circulating biomarkers of metabolic and cardiovascular diseases can help in the early detection and prevention of those diseases. Using proton nuclear magnetic resonance (1H-NMR), we aimed to study the plasma levels of low-molecular-weight metabolites (LMWMs) in a cohort of 307 patients with metabolic diseases to assess their relationships with type-2 diabetes (T2D) and incident atherosclerotic cardiovascular disease (ASCVD).

**Methods:**

We conducted a cross-sectional and prospective study. We included 307 patients attending the Lipid Unit of our University Hospital for the treatment of the following metabolic disturbances and associated disorders: T2D (73.9%), obesity (58.7%), and hypertension (55.1%). 1H-NMR was used to study the plasma levels of 13 LMWMs. LMWM serum concentrations were evaluated in patients with and without T2D. and the correlations with several parameters and their associations with T2D were analyzed. The association between LMWM levels at baseline and the development of ASCVD in patients with T2D after 10 years of follow-up was also evaluated.

**Results:**

Among the LMWMs measured, the branched-chain amino acids (BCAAs) valine, leucine and isoleucine showed a positive association with several clinical and lipid-related biochemical parameters and inflammatory markers (p < 0.05). Likewise, these three BCAAS were associated with diabetes even after adjusting for covariates (p < 0.05). During the follow-up period of 10 years, 29 of the 185 patients with diabetes at baseline (15.68%) developed ASCVD. After adjusting for clinical covariates, baseline levels of valine and alanine were associated with the development of ASCVD (p < 0.05).

**Conclusion:**

Overall, our results indicated that plasma levels of LMWMs measured by 1H-NMR could be potential biomarkers associated with T2D. Moreover, alanine and valine can help in the early detection of the cardiovascular risk associated with this metabolic disease.

**Supplementary Information:**

The online version contains supplementary material available at 10.1186/s12933-023-01958-6.

## Introduction

The identification of biomarkers that can improve the early detection and prevention of metabolic diseases and the accompanying cardiovascular risk has important clinical value. Metabolomic profiling is based on the identification of intermediate molecules and metabolism end-products, which include lipids, lipoproteins, fatty acid species, amino acids, and glycoprotein acetyls [1, 2]. It has emerged as a potential method for identifying new disease-risk biomarkers and offers novel perspectives on the molecular mechanisms, biological processes, and disease pathways implicated [3]. In the field of metabolomic profiling, serum proton nuclear magnetic resonance (1H-NMR) spectroscopy allows for the simultaneous evaluation of a large number of circulating metabolites in a fast and reproducible manner [4]. To date, we and others have demonstrated this tool to be a valid and robust method to assess inflammation in different diseases with an underlying metabolic mechanism [5–7]. Following this approach, metabolomics assessed from a simple blood draw could serve as a clinical approach to improve the detection of highly prevalent diseases.

Diabetes affects more than 500 million people in 2021 and is a major cause of mortality, causing more than 6 million deaths, and these numbers are expected to increase in the upcoming decades [8]. These data show the urgent need for effective prevention of the disease. Type-2 diabetes (T2D) accounts for 90% of the total diabetes cases, and its incidence is growing as a result of an increase in sedentary lifestyle and adiposity [9]. Numerous risk factors, both nonmodifiable (age and genetics) and modifiable (environment, including lifestyle), have been linked to T2D. Each of the following risk factors—diet, body mass index (BMI), physical activity, alcohol consumption, and smoking—has been linked to the risk of diabetes [10]. In fact, these factors account for more than 60% of the disease’s development, with genetic variation accounting for a smaller percentage [11]. The underlying metabolic alterations triggered by these risk factors are closely associated with several chronic diseases, including cardiovascular disease [12]. In fact, atherosclerotic cardiovascular disease (ASCVD), including myocardial infarction, stroke, and peripheral arterial disease, is two times more likely to occur in patients with T2D [13] and is the leading cause of morbidity and mortality globally [14]. For ASCVD risk, blood biomarkers such as cholesterol and low-density lipoproteins (LDL) are established clinical predictors [15]. Given that epidemiological and pathological data consider T2D a major risk factor for ASCVD, the detection of blood biomarkers that add clinical relevance to the factors already known to be implicated in the detection of these common diseases which share a metabolic basis is of potential interest.

Traditional biomarkers of T2D-mediated organ damage rely on distal events in the pathogenesis of the disease or consider, at most, a few biochemical pathways, while the disease involves the deregulation of several molecular pathways over a long period of time [16]. Alternatively, using metabolomic profiling, a large number of biomarkers from various metabolic pathways may be captured in a single measurement. This tool has been effectively used to describe specific metabolites or groups of metabolites linked to various phases of the progression of T2D [17]. For instance, a meta-analysis of 19 prospective studies found that branched-chain amino acids (BCAAs) and aromatic amino acids were linked to both prediabetes and T2D [18]. In contrast, with some metabolites being linked to future ASCVD detection, there is limited research into the identification of circulating metabolites that might predict the onset of ASCVD, particularly in patients with T2D [19].

In the present study, we aimed to measure the plasma levels of different low-molecular-weight metabolites (LMWMs) using 1H-NMR in a cohort of patients with metabolic disorders to assess their relationship with diabetes and incident ASCVD. To test this hypothesis, we cross-sectionally studied the correlations of these LMWMs with different clinical and biochemical parameters as well as their associations with diabetes; in addition, we studied baseline levels of LMWMs in patients with T2D and their association with the development of ASCVD after a 10-year follow-up, providing novel insights into the identification of markers using a modern, high-throughput method that can provide information about future events in this high-risk group.

## Materials and methods

### Study population and study design

We conducted a baseline cross-sectional study and a prospective study with a 10-year follow-up.

We recruited 307 participants for our cross-sectional study who were willing to participate. Participants came to our University Hospital’s Lipid Unit due to hyperlipaemia and metabolism-related disturbances such as obesity, T2D, hypertension, and metabolic syndrome (Met-S). Using standard clinical criteria, obesity [when body mass index (BMI) ≥ 30 kg/m^2^], T2D [when fasting plasma glucose levels ≥ 126 mg/dL and/or glycated hemoglobin (HbA1c) ≥ 6.5% or on hypoglycemic medication] [20], hypertension (when systolic blood pressure ≥ 140 and/or diastolic blood pressure ≥ 90 mm Hg or under antihypertensive drugs) [21], and Met-S following the Adult Treatment Panel III (ATPIII) were identified [22]. ASCVD was defined as myocardial infarction, ischemic stroke, or peripheral artery disease. Subjects with chronic kidney, lung, or cancer diseases were excluded. Patients receiving lipid-lowering drugs underwent a washout period of 6 weeks (8 weeks if they were on fibrates). At the time of examination, information on anthropometry, anamnesis, and physical examination were assessed. Carotid ultrasound was performed to assess subclinical atherosclerosis (intima media thickness and the presence of atherosclerotic plaques). Fasting blood samples were also obtained for standard biochemical analyses and for metabolomic profiling using 1H-NMR.

We conducted a 10-year follow-up of the patients for incident ASCVD. In this part of the study, we looked at the baseline clinical, anthropometric, biochemical, and metabolomic data of patients with or without incident ASCVD who were disease-free at baseline. Then, in 185 patients with diabetes and no prior ASCVD at baseline, we studied the associations between the baseline metabolomic profile and incident ASCVD after 10 years of follow-up.

This study, which followed the guidelines of the Helsinki Declaration, was approved by the Ethical and Clinical Investigation Committee of the Pere Virgili Institute for Health Research (IISPV). A written consent form was signed by all participants.

### Clinical and standard biochemical determinations

Complete anamnesis and anthropometric data, including sex, age, clinical history, and medication, were entered into our database after physical examination. Weight and height values were used to determine the BMI. The carotid intima-media thickness (cIMT) of the right and left common carotid arteries was assessed using a MyLab 60-X Vision sonographer (Esaote, Genova, Italy), and the mean cIMT was determined by averaging the readings from the two carotid arteries. cIMT more than 1.5 mm or protrusions into the lumen that were 50% thicker than the cIMT around them were classified as plaques. Blood samples taken from each participant were prepared for storage at −80 °C in our center’s BioBank prior to usage. Standard biochemical parameters including lipids, apolipoproteins, glucose, and high-sensitivity C-reactive protein (hsCRP) were measured utilizing colorimetric, enzymatic, and immunoturbidimetric assays (Spinreact SA, Spain; Horiba SA, Spain), which were adapted for the Cobas Mira Plus Autoanalyzer (Roche Diagnostics, Spain). Plasma levels of insulin were determined with enzyme-linked immunosorbent assays (ELISAs) following the corresponding manufacturer’s instructions (Mercodia AB, Uppsala, Sweden). Insulin resistance was calculated using the homeostasis model assessment of insulin resistance (HOMA-IR): [fasting insulin (µU/mL) × fasting glucose (mg/dL)/405] [23].

### Clinical follow-up

All patients were seen once a year for medical control and therapy adjustment. Incidental ASCVD was recorded. ASCVD incidence was defined as the presence of acute coronary syndrome or ischemic stroke needing hospitalization and with an unequivocal diagnosis in the discharge report. The incident peripheral vascular disease was defined as the presence of clinical intermittent claudication, ABI < 0.9 and imaging studies showing vascular affection.

### Metabolomic profiling by 1H-NMR

Frozen serum samples were shipped on dry ice to Biosfer Teslab (Reus, Spain) for high-throughput NMR spectroscopy to measure the plasma concentrations of glycoproteins and up to thirteen different LMWMs using a Brucker Avance 600 spectrometer (Bruker GmbH, Germany). Glycoprotein analysis was performed following previously published protocols [5]. In summary, the area of glycoprotein-A (Glyc-A), a chronic inflammatory composite marker, was measured and translated to its concentration. For the analysis of aqueous LMWM quantification by NMR-based metabolomics, an adaptation of Dolphin [24] was used to identify and quantify a target set of LMWMs in the Carr‒Purcell‒Meiboom‒Gill (cpmg) spectra. NMR spectroscopy can generally absolutely quantify a set of ~ 20 LMWM in plasma/serum by using chemical analytical methods. For this study, we selected those metabolites that were present (over the detection limit) and able to be profiled (not overlapped with other metabolites or solvents) in all the patients included. From all the NMR-visible metabolites, 13 met these conditions: acetate, 3-hydroxybutyrate, alanine, creatine, glycine, lactate, tyrosine, glutamate, glutamine, histidine, valine, leucine, and isoleucine. Briefly, serum samples were diluted with 50 mM pH 7.4 phosphate buffer solution and deuterated water prior to 1H-NMR measurement. Then, using previously optimized experimental conditions for one-dimensional 1H-NMR pulse experiments, the spectra were captured at 310 K on a Bruker Advance III 600 spectrometer at a proton frequency of 600.20 MHz. Finally, by checking for all of its resonances in the spectra, each metabolite was identified and then quantified using line-shape fitting [25].

### Statistical analysis

To determine normality, the Kolmogorov‒Smirnov test was applied. Categorical variables are expressed as percentages for the clinical data, and continuous variables are presented as medians with 25th and 75th percentiles (IQR) with nonnormal distributions or as means and standard deviations (SD) when the data are normally distributed. For categorical variables, the chi-square (χ^2^) test was used to examine differences between groups; the t test and Mann‒Whitney U test were applied for continuous variables when corresponding. Spearman’s test, partial correlation, and univariate regression analysis were used to analyse associations between variables. Multivariate linear regression, including age, sex, BMI, systolic blood pressure (SBP), cholesterol, and triglycerides as covariates, was also used to study adjusted associations. All statistical analyses were performed using IBM SPSS Statistics (version 28.0.0.0, Madrid, Spain). Results were considered statistically significant when the associated p value was less than 0.05.

## Results

### Cross-sectional study

In our study, 307 participants were included with a median age of 60 (51–65) years and 49.5% were female. Obesity, T2D, and hypertension were present in 58.7%, 73.9%, and 55.1% of the individuals, respectively. Additionally, ASCVD was present in 13.4% of the patients, and atherosclerotic plaques were present in 37.4%. Table 1 summarizes the patient’s clinical, anthropometric, and biochemical details.

**Table 1:** Clinical, anthropometric, and biochemical characteristics of the 307 study participants

Number of participants	307
Age, sex, and clinical data	
Age, years	60 (51–65)
Women, %	49.5
Obesity, %	58.7
Type 2 diabetes, %	73.9
Hypertension, %	55.1
ASCVD, %	13.4
Plaque, %	37.4
cIMT, mm	0.69 (0.64–0.78)
Physical measurements	
BMI, kg/m^2^	30.7 (28.28–35.78)
Waist circumference (cm)	104 (97–113)
Systolic BP, mmHg	138 (128–150)
Diastolic BP, mmHg	80 (75–87)
Biochemical data	
Total cholesterol, mmol/L	5.64 (4.88–6.83)
Triglycerides, mmol/L	1.86 (1.24–3.08)
VLDL-C, mmol/L	0.9 (0.56–1.32)
LDL-C, mmol/L	3.54 ± 1.11
HDL-C, mmol/L	1.11 (0.93–1.32)
Non-HDL-C, mmol/L	4.46 (3.78–5.52)
Apo B-100, mg/dL	118 (98–141)
Apo A-I, mg/dL	121 (105–138)
Glucose, mg/dL	133 (107–168)
hsCRP, mg/L	2.23 (1.26–3.75)
Glyc-A, µmol/L	895.14 (748.45–1099.62)

Data are percentages (%) for categorical variables, medians (interquartile ranges) for nonparametric continuous variables or means ± SDs for continuous normally distributed variables

*ASCVD* atherosclerotic cardiovascular disease, *cIMT* carotid intima-media thickness, *BMI* body mass index, *systolic BP* systolic blood pressure, *diastolic BP* diastolic blood pressure, *VLDL-C* very low-density lipoprotein cholesterol, *LDL-C* LDL cholesterol, *HDL-C* high-density lipoprotein cholesterol, *Non-HDL-C* non-HDL cholesterol, *Apo B-100* apolipoprotein B100, *Apo A-I* apolipoprotein A1, *hsCRP* high-sensitivity C-reactive protein, *Glyc-A* glycoprotein A

#### Associations of NMR-measured plasma LMWMs with clinical, biochemical, and inflammatory markers

The majority of the thirteen NMR-measured LMWMs showed significant associations with several clinical, lipid-related, glycemic, and inflammatory markers (Table 2). The BCAAs valine, leucine, and isoleucine showed associations with all of the variables (p < 0.05), with the exception of cIMT, diastolic BP, and apoA-I. Additionally, lactate exhibited significant positive correlations (p < 0.05) with a wide range of variables, while glutamine only showed significant negative correlations (p < 0.05). Furthermore, it was observed that the plasma values of the three BCAAs, acetone, 3-hydroxybutyrate, alanine, creatine, lactate, and histidine were different between patients when grouped by the presence or absence of T2D (Additional file 1: Table S1).

**Table 2:**
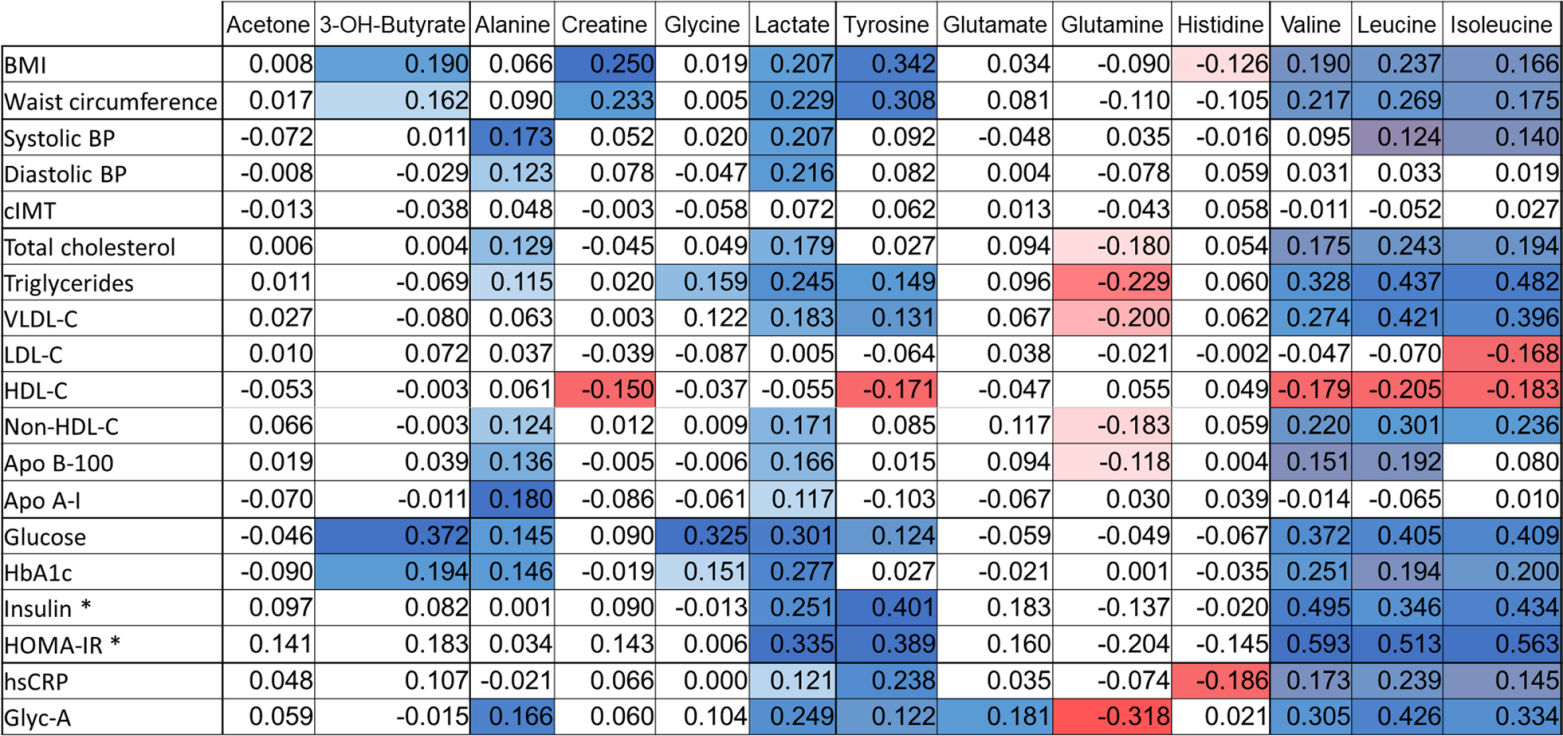
Correlations of LMWMs with clinical, lipidic, glycemic, and inflammatory markers in the study population

Partial correlations adjusted for age and sex. Significant correlation values (p < 0.05) are represented in a blue‒red scale, with blue tones representing positive correlations and red tones representing negative correlations; the darkest tones indicated stronger positive or negative correlations

*3-OH-butyrate* 3-Hydroxybutyrate, *cIMT* carotid intima-media thickness, *BMI* body mass index, *systolic BP* systolic blood pressure, *diastolic BP* diastolic blood pressure, *VLDL-C* VLDL cholesterol, *LDL-C* LDL cholesterol, *HDL-C* HDL cholesterol, *non-HDL-C* non-HDL cholesterol, *Apo B-100* apolipoprotein B100, *Apo A-I* apolipoprotein A1, *HbA1c* glycated hemoglobin, *HOMA-IR* homeostasis model assessment of insulin resistance, *hsCRP* high-sensitive C-reactive protein, *Glyc-A* glycoprotein A

^a^n = 74

#### Associations of NMR-measured plasma LMWMs with T2D

**Fig. 1:**
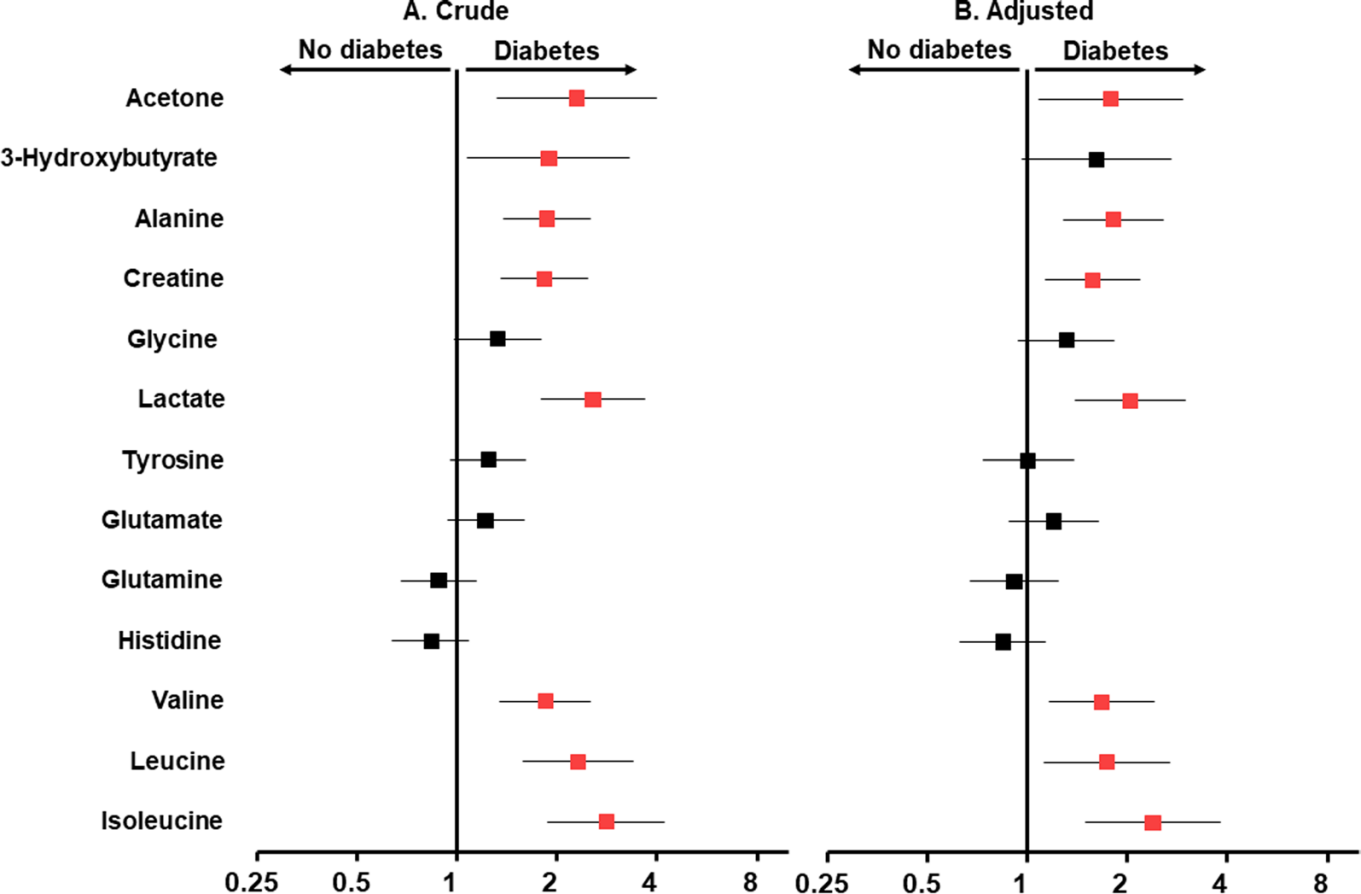
Associations between plasma LMWMs and T2D. Data are presented as the odds ratios (OR) per 1-SD-deviation-higher metabolic biomarker. Squares represent the OR, and horizontal lines represent the corresponding 95% CI. **A** Crude univariate associations are shown and **B** analyses adjusted for age, sex, SBP, BMI, plasma cholesterol, and triglycerides. Red squares indicate variables that are significantly associated with T2D (p < 0.05). The vertical line marks an OR of 1, indicating no effect on T2D

Associations with T2D were found with eight out of the thirteen NMR-measured LMWMs, and both the crude and adjusted odds ratios (OR) are presented in Fig. 1. The following variables had significant positive associations with T2D after adjustment for clinical and biochemical covariates: acetone (adjusted OR [95% CI] 1.78 [1.08–2.93]), alanine (1.81 [1.28–2.56]), creatine (1.57 [1.13–2.18]), lactate (2.04 [1.4–2.99], valine (1.67 [1.16–2.4]), leucine (1.73 [1.12–2.68]), and isoleucine (2.39 [1.5–3.8]).

### Prospective study

After a 10-year follow-up, we collected data on the development of ASCVD in 260 patients in our cohort with no clinical history of previous ASCVD at baseline. In this period, ASCVD was reported in 32 patients (12.31% incidence). In addition, an incidence of 15.68% was reported for patients with T2D at baseline, 14.29% for patients with obesity, and 18.8% for patients with hypertension.

#### Baseline plasma levels of LMWMs and incidence of ASCVD

Table 3 summarizes the baseline clinical, anthropometric, biochemical, and metabolomic data of the 260 patients included in the follow-up. Higher percentages of patients with diabetes (91%), hypertension (78%), or plaques (56%) at baseline were observed in the 32 patients who developed ASCVD (p < 0.05). Baseline systolic and diastolic BP and glucose were higher in the patients who developed ASCVD (p < 0.05). The NMR-measured metabolomic profile of plasma LMWMs showed increased levels of alanine, valine, and isoleucine (p < 0.05) in the group that had developed ASCVD (p < 0.05, not shown).

**Table 3:** Baseline clinical and metabolomic profiles of the patients according to ASCVD after a 10-year follow-up

ASCVD at 10 years			
	No	Yes	p values
Number of participants	228	32	
Age, sex, and clinical data			
Age, years	57 (50–65)	63 (55–65)	0.056
Women, %	50	34.4	0.098
Obesity, %	55.5	65.6	0.279
Type 2 diabetes, %	68.4	90.6	**0.009**
Hypertension, %	48.2	78.1	**0.002**
Plaque, %	33.2	56.3	**0.011**
Physical measurements			
BMI, kg/m^2^	30.52 (28–34.66)	31.4 (28.95–35.84)	0.278
Waist circumference (cm)	103 (95–112)	108.5 (99–113)	0.169
Systolic BP, mmHg	135 (125–146)	149 (136–163)	**< 0.001**
Diastolic BP, mmHg	80 (73–85)	88 (79–93)	**0.002**
Biochemical data			
Total cholesterol, mmol/L	5.65 (4.84–6.7)	5.54 (4.88–6.6)	0.841
Triglycerides, mmol/L	1.86 (1.22–2.88)	2.63 (1.37–3.98)	0.067
VLDL-C, mmol/L	0.89 (0.53–1.29)	1.1 (0.63–1.69)	0.156
LDL-C, mmol/L	3.54 ± 1.12	3.24 ± 1.31	0.166
HDL-C, mmol/L	1.11 (0.93–1.33)	1.11 (0.89–1.33)	0.665
Non-HDL-C, mmol/L	4.45 (3.74–5.53)	4.5 (3.72–5.46)	0.726
Apo B-100, mg/dL	118 (96–141)	113 (100–132.5)	0.812
Apo A-I, mg/dL	120 (106–138)	122.5 (108.5–143)	0.595
Glycemia			
Glucose, mg/dL	128.5 (103–162)	142.5 (121.5–173.5)	**0.033**
HbA1c, %	6.3 (5.5–7.5)	6.1 (5.6–7.1)	0.812
Insulin, mU/L	11.54 (7.83–22.42)	17.95 (16.45–20.83)	0.266
HOMA-IR	3.25 (1.9–6.81)	7.35 (5.1–8.54)	0.146
Inflammatory markers			
hsCRP, mg/L	2.17 (1.28–3.64)	2.55 (1.28–3.56)	0.753
Glyc-A, µmol/L	889.6 (734.53–1084.18)	969.82 (768–1160.07)	0.156

Data are percentages (%) for categorical variables, medians (interquartile ranges) for nonparametric continuous variables or means ± SDs for continuous normally distributed variables. Patients were grouped as atherosclerotic cardiovascular disease (ASCVD)-free (No) or confirmed ASCVD (Yes). P values from χ^2^ for categorical variables, t test, or Mann‒Whitney U test for continuous variables. Bold values indicate p < 0.05

*cIMT* carotid intima-media thickness, *BMI* body mass index, *systolic BP* systolic blood pressure, *diastolic BP* diastolic blood pressure, *VLDL-C* VLDL cholesterol, *LDL-C* LDL cholesterol, *HDL-C* HDL cholesterol, *non-HDL-C* non-HDL cholesterol, *Apo B-100* apolipoprotein B100, *Apo A-I* apolipoprotein A1, *HbA1c* glycated hemoglobin, *HOMA-IR* homeostasis model assessment of insulin resistance, *hsCRP* high-sensitivity C-reactive protein

#### Associations of baseline plasma LMWM with ASCVD in patients with T2D

**Fig. 2:**
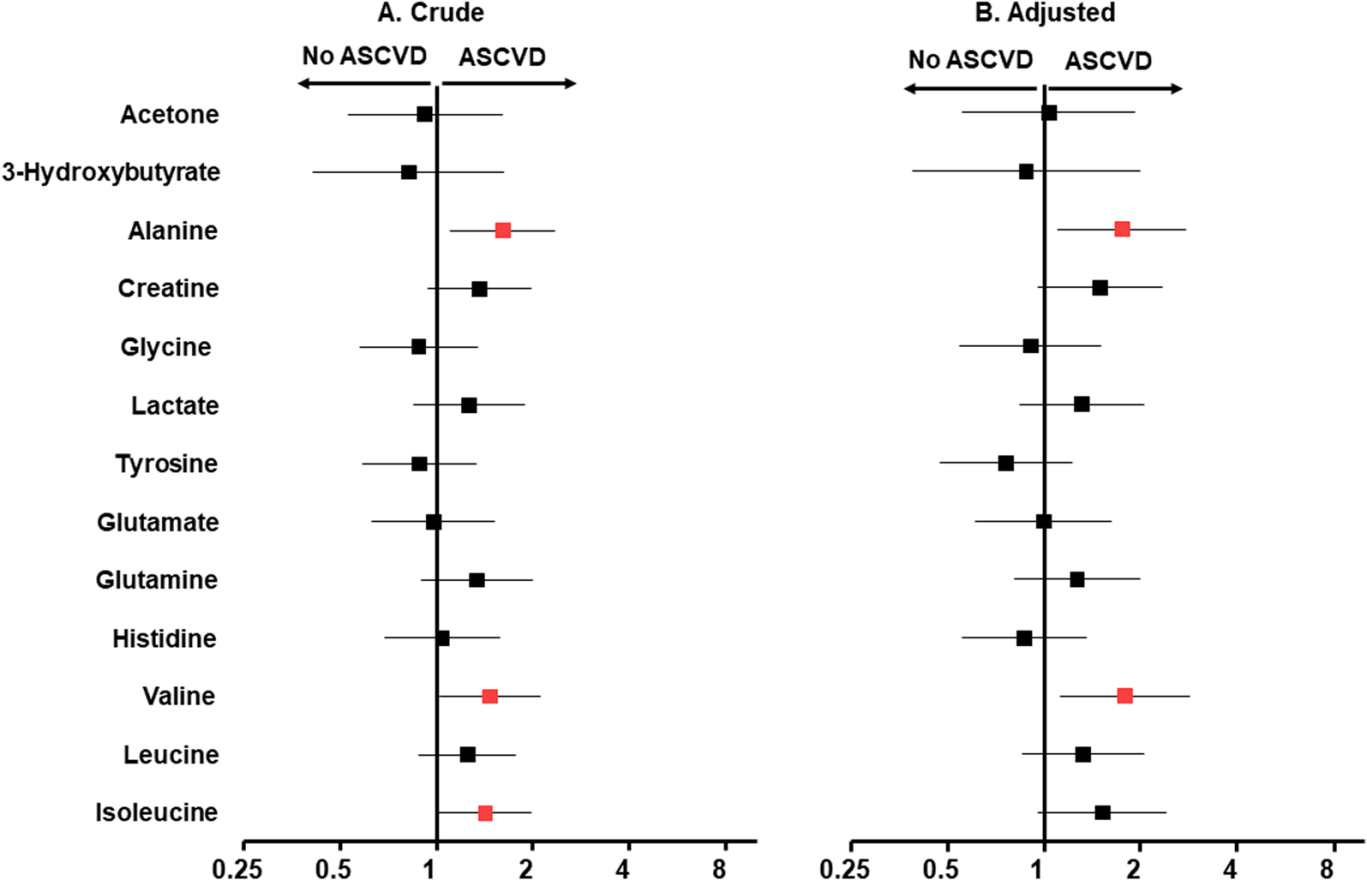
Associations between baseline plasma LMWMs and ASCVD in patients with T2D. Data are presented as the odds ratios (OR) per 1-SD-deviation-higher metabolic biomarker. Squares represent the OR, and horizontal lines represent the corresponding 95% CI. Crude univariate associations are shown in the left panel, and analyses adjusted for age, sex, SBP, BMI, plasma glucose, cholesterol, and triglycerides are shown in the right panel. Red squares indicate those with a significant association (p < 0.05). The vertical line marks an OR of 1, indicating no effect on incident ASCVD

In this prospective analysis, 185 patients with T2D at the beginning of the study were selected, and the baseline levels of the NMR-measured LMWMs were analyzed. Twenty-nine patients (15.68%) were reported as new cases of ASCVD were reported after 10 years. Baseline levels of alanine (OR: 1.61 [1.11–2.35]), valine (1.47 [1.02–2.11]), and isoleucine (1.42 [1.02–1.98]) were shown to have significant positive associations with the development of ASCVD (Fig. 2), of which the associations with alanine and valine remained significant after adjusting for covariates (p < 0.05).

## Discussion

In the present study, we cross-sectionally investigated the association between plasma levels of thirteen LMWMs assessed by 1H-NMR and diabetes in a cohort of patients with hyperlipidemia at who were at metabolic risk. We found significant positive correlations between BCAAs and several clinical, lipid-related, glycemic, and inflammatory markers. Likewise, these three BCAAs remained associated to T2D even after controlling for clinical cofounders. During the follow-up period of 10 years, 29 of the 185 patients with T2D at baseline developed ASCVD. In this prospective analysis, baseline levels of alanine and the BCAA valine were linked to the development of ASCVD.

Mechanistically, BCAAs have been previously reported to impact metabolic health by their possible contribution to insulin resistance [26]. Several studies revealed higher plasma BCAA levels in humans with insulin resistance, T2D, and T2D-related metabolic disturbances [27, 28]. Another human study linked higher levels of BCAAs and inflammation with genetically predicted insulin resistance, suggesting that BCAA metabolism may lie in a causal pathway from adiposity and insulin resistance to T2D [29]. In a study conducted by Newgard et al. [30] in both rats and humans, it was reported that an excess of BCAA catabolism in obese individuals may be a reflection of alterations in certain amino acid metabolites linked to insulin resistance, and BCAAs may independently contribute to the development of insulin resistance and diabetes in the context of a poor dietary pattern that includes high fat consumption. In this context, it was shown how rats fed a high-fat diet supplemented with BCAAs, despite having reduced food intake and weight gain equivalent to standard chow-fed animals, were as insulin-resistant as rats fed a high-fat diet alone. At the molecular level, these associations are likely to be due to the activation of mTORC1 or due to the dysmetabolism of BCAAs, whose metabolite accumulation results in metabolic dysfunction associated with T2D [26], with both hypotheses in need of further research to fully understand their relative contributions. Other factors, such as inflammation, may also play a role in the relationship between BCAAs, insulin resistance, and diabetes, since we found how these metabolites correlate with NMR-measured inflammatory markers that have been previously shown to be increased in dysmetabolic patients [5]. These possible mechanisms are in line with our results, as we showed that BCAA are positively correlated and associated with lipidic, glycemic, and inflammatory markers.

We also found that the pyruvate-generating metabolites alanine, creatine, and lactate were associated with diabetes, with lactate showing positive correlations with most of the variables assessed. The relationship with lactate accumulation has been previously documented [31, 32] based on the fact that lactate accumulation causes acidosis that leads to several adverse effects. Alanine, one of the main gluconeogenic precursors, can be transformed into the intermediates glutamate and pyruvate, which function as important links between the metabolism of carbohydrates and amino acids [33]. Overall, the detection of altered levels of intermediates of different metabolic routes may be a sign of an unbalanced metabolism, which may help to explain the associations found between LMWMs and diabetes.

We also addressed the possible predictive values of these molecules following a prospective approach. Our data indicate that baseline levels of alanine and valine are associated with the development of ASCVD in patients who had diabetes at baseline. In accordance, the predictive potential of BCAAs in diabetes has been previously described [34, 35]. A large cohort study including > 50,000 individuals from UK Biobank, with incident T2D in 1719 of the participants assessed during 12 years of follow-up, revealed that the addition of 143 plasma NMR-measured metabolites, including BCAAs, to an existing risk prediction model comprising basic clinical parameters provided added value for the prediction of T2D risk [36]. Following these observations, another prospective cohort study of > 1500 individuals in the Chinese population with an average follow-up period of 8 years also reported that the inclusion of 31 metabolites improved risk estimation and T2D prediction, with BCAAs being among the metabolites with stronger associations [37]. An updated meta-analysis including 61 prospective cohort reports on T2D risk highlighted not only robust associations with BCAAs but also other amino acids and, remarkably, a set of lipid metabolites [38]; that analysis evaluated the association of metabolite concentrations not only in plasma or serum but also urine as another biological sample to be analyzed by high-throughput metabolomic platforms.

For cardiovascular risk assessment, to date, lipid species and their metabolic pathways are the main targets when profiling ASCVD; thus, alterations in various families such as phospholipids, sphingomyelins, or fatty acids, have been reported to be related to future cardiovascular events [39, 40]. In our study, we did not detect significant differences in the lipidic parameters between those with incident ASCVD and those without, since we analyzed a cohort of patients with lipid profiles that were already relatively altered. Additionally, epidemiological studies have demonstrated that elevated plasma BCAA concentrations may be used to identify individuals with heart failure, coronary artery disease or hypertension and can be used to predict ASCVD in these groups [41, 42]. Three cohort-based prospective datasets that included NMR metabolomic profiling were analyzed by Würtz et al., and they identified several metabolites that were associated with ASCVD, including amino acids, fatty acids, and lipids [43]. Baseline levels of BCAAs were associated with incident ASCVD in 2207 US women over a period of 18 years follow-up even after adjusting for the established ASCVD risk factor LDL-C [44]. Given the global burden of metabolic diseases and cardiometabolic risk, ASCVD is associated with T2D, causing at least half of the mortality in those patients with T2D, with coronary artery disease and stroke being significant contributors to comorbidity and mortality among patients [45, 46]. In this line, as metabolomic methods for understanding the metabolic basis of ASCVD increase [47, 48], the search for biomarkers to identify those patients at greatest risk using platforms such as 1H-NMR is now a challenge. This includes the development of ASCVD in patients with T2D that we have addressed in the present study.

It is important to note some limitations of our study. First, our findings are based on associations and correlations, so the explanation of potential causative molecular pathways is limited. For the detection of insulin resistance, although not a gold standard, we used HOMA-IR as a surrogate since it allowed us to identify correlations with our NMR-measured biomarkers. In our regression models, we were not able to adjust for antidiabetic medication prescribed to our participants, since a large majority were already patients with T2D at the beginning of the study and could not be left untreated; this might have influenced metabolic profiles. In addition, dietary/nutritional habits were not assessed, and the influence of diet especially on essential amino acids such as BCAAs, could not be evaluated. It should also be noted that since we only have baseline measurements, it is not possible to evaluate variations in LMWMs and other parameters during disease progression; nevertheless, the observed associations of LMWMs with occurrent disease are robust and provide value to our findings. Last, our follow-up study includes a limited sample size, especially in the group of patients who developed ASCVD. However, the 10-year follow-up period strengthens our findings. The use of 1H-NMR to perform a metabolomic profile of the subjects in our study is its main strength. It represents a high-throughput, reliable, and robust technology that reflects the molecular signature of the patients, providing insights into altered metabolites and their possible roles in the underlying mechanisms of disease progression. Future research should focus on verifying these findings in larger cohorts and clarifying the molecular mechanisms implicated.

In conclusion, we measured a set of thirteen LMWMs in a cohort of patients at cardiovascular risk using 1H-NMR, and we reported correlations with several clinical, lipidic, glycemic, and inflammatory markers as well as additional associations with T2D, especially BCAAs. We also provide evidence that the measurement of baseline LMWM levels is associated with the future development of ASCVD in patients with T2D after 10 years. Our findings suggest that metabolomics-based technologies can help in the early detection of cardiovascular risk in patients with T2D, even though the causal effect of these metabolites on the cardiometabolic system remains unknown.

### Supplementary Information

**Additional file 1:** **Table S1.** Plasma values of the NMR-measured LMWM of the cohort grouped by the presence or absence of T2D.

## Data Availability

Upon request, the authors will provide a data collection that contains the raw data used to support the findings of this study.
