# Supplementary material for: Serum branch-chained amino acids are increased in type 2 diabetes and associated with atherosclerotic cardiovascular disease
Source: Cardiovasc Diabetol. 2023 Sep 14;22:249. doi: 10.1186/s12933-023-01958-6 (PMC10503204; doi:10.1186/s12933-023-01958-6)
Supplement: Supplementary file 1 — Additional file 1: Table S1. Plasma values of the NMR-measured LMWM of the cohort grouped by the presence or absence of T2D. [file 12933_2023_1958_MOESM1_ESM.docx]

|  | **Type 2 diabetes (T2D)** | |  |
| --- | --- | --- | --- |
|  | **No** | **Yes** |  |
| Number of partipants | 80 | 227 | p values |
| **NMR-measured LMWM** |  |  |  |
| Acetone, mmol/L | 0.008 (0.006-0.01) | 0.012 (0.009-0.016) | **<0.001** |
| 3-Hydroxybutyrate, mmol/L | 0.079 (0.064-0.097) | 0.099 (0.079-0.134) | **<0.001** |
| Alanine, mmol/L | 0.329 (0.305-0.373) | 0.36 (0.319-0.407) | **<0.001** |
| Creatine, mmol/L | 0.055 (0.044-0.065) | 0.066 (0.052-0.079) | **<0.001** |
| Glycine, mmol/L | 0.119 ± 0.045 | 0.134 ± 0.056 | 0.063 |
| Lactate, mmol/L | 0.855 (0.713-1.049) | 1.075 (0.885-1.351) | **<0.001** |
| Tyrosine, mmol/L | 0.036 ± 0.12 | 0.039 ± 0.01 | 0.101 |
| Glutamate, mmol/L | 0.269 ± 0.048 | 0.279 ± 0.051 | 0.138 |
| Glutamine, mmol/L | 0.163 (0.118-0.195) | 0.1429 (0.103-0.194) | 0.149 |
| Histidine, mmol/L | 0.06 (0.049-0.071) | 0.056 (0.046-0.064) | **0.040** |
| Valine, mmol/L | 0.197 (0.174-0.222) | 0.217 (0.197-0.242) | **<0.001** |
| Leucine, mmol/L | 0.092 (0.075-0.107) | 0.106 (0.09-0.122) | **<0.001** |
| Isoleucine, mmol/L | 0.027 (0.019-0.032) | 0.034 (0.027-0.04) | **<0.001** |

**Additional file 1: Table S1. Plasma values of the NMR-measured LMWM of the cohort grouped by the presence or absence of T2D.**

*Data are median (interquartile range) for non-parametric continuous variables or means ± SD for continuous normally distributed variables. Patients were grouped as T2D-free (No) or confirmed T2D (Yes). P values from t test or Mann‒Whitney U test for continuous variables. Bold values indicate p<0.05.*
